# Effects of auditory and socio-demographic variables on discontinuation of hearing aid use among older adults with hearing loss fitted in the Chilean public health sector

**DOI:** 10.1186/s12877-019-1260-6

**Published:** 2019-09-03

**Authors:** Eduardo Fuentes-López, Adrian Fuente, Gonzalo Valdivia, Manuel Luna-Monsalve

**Affiliations:** 1 Departamento de Ciencias de la Salud, Carrera de Fonoaudiología, Facultad de Medicina, Pontificia Universidad Católica de Chile, Avenida Vicuña Mackenna 4860, Macul, Santiago, Chile; 2 École d’orthophonie et d’audiologie, Faculté de médecine, Université de Montréal, Montréal, Québec Canada; 3 Centre de recherche de l’Institut universitaire de gériatrie de Montréal, Montréal, Québec Canada; 4 Departamento de Salud Pública, Facultad de Medicina, Pontificia Universidad Católica de Chile, Santiago, Chile; 5 Escuela de Fonoaudiología, Facultad de Ciencias de la Salud, Universidad San Sebastián, Concepción, Chile

**Keywords:** Hearing aid use, Older adults, Socio-demographic variables

## Abstract

**Background:**

The percentage of older adults with hearing loss who stop using their hearing aids and the variables associated with this phenomenon have not been systematically investigated in South America. This problem is relevant to the region since countries such as Colombia, Brazil and Chile have public programmes that provide hearing aids to older adults. The aims of this study were to determine the percentage of older adults fitted with a hearing aid at a public hospital in Chile who subsequently stop using it and the auditory and socio-demographic variables associated with the hazard of discontinuing hearing aid use.

**Methods:**

A group that included 355 older adults who had been fitted with a hearing aid was studied retrospectively. In a structured interview, participants were asked about socio-demographic variables and answered part of the Chilean National Survey on Health, evaluating self-perceived hearing loss and responding to questions about discontinuation of hearing aid use and their satisfaction with the device. Survival models were applied to determine the hazard of stopping hearing aid use in relation to the variables of interest.

**Results:**

The rate of discontinuation of hearing aid use reached 21.7%. Older adults stopped using their hearing aids mainly during the first 5–6 months post-fitting, and then this number steadily increased. The income fifth quintile was 2.56 times less likely to stop using the hearing aid compared to the first. Those who self-reported that they could not hear correctly without the hearing aid were 2.62 times less likely to stop using it compared to those who reported normal hearing. The group that was very dissatisfied with the hearing aid was 20.86 times more likely to discontinue use than those who reported satisfaction with the device.

**Conclusions:**

Socio-demographic variables such as economic income and auditory factors such as self-perceived hearing loss and satisfaction with the device were significantly associated with the hazard of stopping hearing aid use. Self-perceived hearing loss should be considered part of the candidacy criteria for hearing aids in older adults in Chile and other (developing) countries.

## Background

It has been reported that between 1 and 40% of older adults from developed countries stop wearing their hearing aids post-fitting [1–4]. In a recent systematic review, Ng and Loke [5] identified the degree of hearing loss, type of hearing aid (i.e. more or less sophisticated), tolerance for background noise while listening to speech, and hearing aid adjustment to better match prescription targets as the audiological variables associated with adoption and use of hearing aids in older adults. Non-audiological variables associated with these outcomes included self-perceived hearing problems, expectations, gender and income levels. These results are similar to the results of another systematic review conducted by Knudsen et al. [6] A scoping review carried out by McCormack and Fortnum [7] about the reasons for non-use of hearing aids found that perceived benefit, fit and comfort of the hearing aid (including difficulties with handling the device and adverse effects); maintenance and other device-related variables (e.g. cost of repairs and batteries, malfunction, feedback issues); and negative attitudes and appearance (i.e. stigma and cosmetic concerns) were the variables associated with discontinuation of the use of hearing aids.

Despite the large number of studies of factors associated with hearing aid use and/or discontinuation of their use in older adults from developed countries [5–8], very little is known from developing countries. Due to a number of factors such as cost of the devices and access to hearing health care professionals along with cultural differences, the results from developed countries cannot be directly extrapolated to the context of developing countries. This is a major issue considering that two thirds of the world’s older adults live in developing economies [9]. Due to the growing number of older adults and the high prevalence of hearing loss in this population, government programmes in some South American countries such as Brazil [10], Colombia [11] and Chile [12] provide hearing aids for older adults with hearing loss. However, little is known about the success of such programmes (i.e. how long older adults continue using their hearing aids post-fitting).

Specifically in Chile, since 2007, adults aged 65 and older who require hearing aids are provided with one device (if they are users of the public healthcare system) either for free or with a maximum co-payment of 20% of the device price [13]. The latter is determined based on the person’s income levels. An ENT doctor must prescribe the device based on pure-tone audiometric results (i.e. pure-tone average [500, 1000, 2000, and 4000 Hz] ≥ 40 dB HL [14], in the better ear). Under this programme, hearing aids with multiple channels and up to four programmes whose cost is low (approximately $105 USD) are provided by public hospitals. Each hospital puts out a tender for the contract to purchase the hearing aids and the company chosen fits them and perform follow-up appointments with the patient (in the majority of cases, this involves three sessions to adjust the hearing aid and teach basic aspects of its care and use).

Despite the fact that the programme that provides hearing aids for older adults in Chile began more than 10 years ago, both the percentage of those who have stopped using them –over the course of time– after the initial fitting and the variables associated with discontinuation are unknown. Although prior studies in South America [15, 16] have estimated the percentage of hearing aid use and shown the influence of socio-demographic variables such as economic income and age, these studies had a cross-sectional design (measuring whether the hearing aid is used or not). This allows for metrics such as prevalence to be estimated, a measurement that does not consider the speed at which the event occurs or the risk of the event appearing over time, such as incidence density and hazard function do, respectively. Therefore, it is not known whether variables such as age, gender or income level are associated with the incidence and/or hazard of discontinuing hearing aid use over time among the population of older adults using the public system in Chile. The same is true for auditory variables (e.g. pure-tone threshold average, self-perceived hearing difficulties, satisfaction with the hearing aid). As mentioned above, a PTA ≥ 40 dB HL in the better ear is considered the main criterion for hearing aid prescription for older adults under this programme [14]. Subjective variables such as self-perceived hearing problems or difficulty hearing in daily life (hearing disability), which may influence older adults to stop using the hearing aid, are not considered for hearing aid prescription. Thus, the aims of this research were to (a) determine the percentage of older adults fitted with a hearing aid at a public hospital in Chile who subsequently stopped using it, and (b) determine the association between auditory (i.e. self-perceived hearing difficulties, pure-tone threshold, auditory disability and hearing aid satisfaction) and socio-demographic variables with regard to the hazard of discontinuation of hearing aid use in the previously mentioned population.

## Methods

### Sample

The sample was comprised of 355 older adults, hearing aid users aged between 65 and 85 years. All participants were monaurally fitted with a hearing aid at La Florida Public Hospital in Santiago, Chile.

The sample size was calculated by determining the effect size based on the data reported by a previous study about discontinuation of hearing aid use in a sample of South Korean adults [8]. The measure of the effect (hazard ratio [HR]) was estimated using the area under the Kaplan-Meier curve obtained in the aforementioned study when two groups of participants (with and without peer support) were compared. According to the times (i.e. years after the hearing-aid fitting, in a maximum follow-up time of 10 years) obtained and the area under the survival curve, the expected value (E) was estimated through the integral of the probability density function and then the HR was calculated. Thus, using this measure of effect (HR = 1.42) with an α = 0.05 and an 80% power for a logrank (with one-tailed test), a total of 206 participants (103 per group) was estimated for crude differences between groups (without covariate adjustment).

In order to reach statistical power for the multivariate survival analyses, which included several covariates, a larger sample size than the above was required. In this study, we explored the association between discontinuation of hearing aid use and 11 variables: gender, age, education level, income level, own health perception, auditory disability, three factors relating to own perception of hearing level, pure-tone average (500, 1000, 2000, 4000 Hz) in the fitted ear and satisfaction with the hearing aid. Simulation studies have established that for regression models for the analysis of survival data, it is recommended to include one predictor variable per 10 events in the sample [17–19]. Taking the South Korean study mentioned above as a reference study, it was reported that 31% of the participants discontinued hearing aid use (i.e. the event). Thus, in the present study, to include 11 variables, it is necessary to have 110 events which should be observed in 355 recruited participants (31% of 355 = 110 events).

### Ethical approval

The research protocol was approved prior to the commencement of the study by La Florida Hospital’s Ethics Committee and by the Scientific Ethics Committee of the Pontifical Catholic University of Chile. All participants signed an informed consent form previously cleared by the boards of the aforementioned institutions.

### Procedures

La Florida Hospital commenced providing hearing aids to older adults under the government programme in 2015. At the time of this research, 823 older adults had been fitted with a hearing aid for the first time at least one year before the study began and thus were considered prospective participants for this research. The list containing this number of participants with their contact details was provided by the hospital authorities. Prospective participants were initially selected at random and then contacted by telephone with the aim to explain the study and invite them to take part in it. Those who agreed to participate and gave consent for the research team to review their medical files were then preselected. Subsequently, their medical records were accessed to determine the possible presence of either external or middle-ear problems not associated with age-related hearing loss. Participants presenting with such problems were excluded. This is because in the presence of such disorders, the hearing aid fitting procedure has to be adapted accordingly [20]. This may imply differences in the gain provided by the hearing aid as compared to problems associated with cochlear dysfunction [20] as observed in age-related hearing loss. In addition, otitis externa has been associated with discontinuation of hearing aid use [7]. Participants who did not present with such problems were then visited at their homes by trained personnel with the aim to collect the data for the study.

The home visit began with a shortened version of the Mini-Mental State Examination (MMSE) that was previously adapted and validated in Chile [21]. In addition, the aforementioned instrument has been used in studies conducted in both Chile [15, 22] and other Latin American countries [23–25]. The maximum overall score for this screening tool is 19. A cognitive impairment was suspected when the person obtained a score less than or equal to 12 points [22]. Therefore, participants with a score less than or equal to 12 were excluded from the study. In addition, participants presenting with oral communication problems (e.g. aphasia) were excluded from the study. Participants with either of these disorders (i.e. cognition and oral communication) were excluded as they are likely to have problems understanding and/or providing responses to the questionnaires used in this study (see below). If a participant was excluded for such reasons, then another person was contacted and visited until the sample of 355 participants was achieved.

Next, part of the Chilean National Survey on Dependency in Older Adults (ENADEAM in Spanish) [22] questionnaire was carried out. This instrument has been previously validated by a panel of experts and used in similar prior studies in Chile [15]. In this questionnaire, age, self-perceived general state of health, years of education and monthly income were obtained. The participants were asked about their last year of education and to indicate how many years of education they had obtained. In the case of monthly income, an open-ended question was asked: *In total, as far as your income is concerned, how much money do you receive on average in a normal month?* The question about self-perceived state of health (*In general, how would you describe your health?*) had five possible answers in a Likert-type format, ranging from *excellent health (1 point)* to *bad health (5 points).*

Afterwards, a question from the Chilean National Health Survey (ENS) [26] was used to ask about self-perceived hearing problems: *If you use a hearing aid, answer the following questions, thinking about your hearing without wearing a hearing aid. Do you think you hear normally in both ears?* Two other questions with a binary response (yes/no) were also asked: *Are you capable of watching a television programme at a volume that is acceptable to other people?* and *Are you capable of following a conversation involving three or more people?*

Auditory disability was evaluated using the Spanish version of the Amsterdam Inventory for Auditory Disability and Handicap (S-AIADH) [27]. This instrument is made up of 30 questions about listening situations in daily life with a Likert-type response format. Each question has four response alternatives: almost never, occasionally, frequently and almost always. Alternatives are rated from 1 (almost never) to 4 (almost always). However, items 18 and 30 are rated from 1 (almost always) to 4 (almost never), as they examine how often the person experiences music as being too loud (item 18) and how often they feel they are missing parts of a melody (item 30). Thus, the greater the score on the S-AIADH, the lesser the auditory disability [27].

Auditory thresholds were obtained using information from the older adults’ medical records. Audiologists at the hospital carried out air conduction pure-tone audiometry from 250 to 8000 Hz in a double-wall, sound-proofed booth according to ISO norm 8253–1. The air conduction pure-tone threshold average (500, 1000, 2000 and 4000 Hz) of the ear fitted with the hearing aid was calculated.

The time that had passed since the participant discontinued the use of the hearing aid was measured beginning when the hearing aid was fitted. The latter date was obtained from hospital records. The date when the participant discontinued hearing aid use was obtained from the participants themselves using the question asked by Bertoli et al. [28]: *Do you use your hearing aid*? The response options – considering use in days per week – included not at all, every day, most days (at least 5 days per week), some days (1–4 days per week) and only occasionally. Among those who selected the ‘not at all’ option, the time that passed before they stopped using the hearing aid was determined by asking the question, *When did you stop using the hearing aid?* To diminish memory bias, the interviewer referred to important biographical facts (for example, birthdays, births and anniversaries) and bank holidays (for example, the Chilean national holiday, Christmas, New Year’s Day). In addition, a multiple-choice question was included from the questionnaire used by Bertoli et al. [28] about the reasons for discontinuation of hearing aid use: *If you never use your hearing aid, please indicate why not*. The response options were no/poor benefit, noisy situations are disturbing, poor sound quality, difficulties using it (for example, controlling the volume), poor fit and comfort, negative side effects (for example, rashes, itching, pain, build-up of wax), no need and other reasons. In this same study, the question used to measure satisfaction with the hearing aid among those still using it was *Are you satisfied with your hearing aid?* The four response options provided ranged from very satisfied (1 point) to very dissatisfied (4 points).

All questions/statements and possible replies were read aloud by the interviewer to older adults with mediocre or poor eyesight, even when they wore glasses, giving them a chance to ask for clarification of any doubts they might have. The response options of the instruments were in print form, with a letter size large enough to read easily (Arial font, size 40). Older adults could give their answers either verbally or by pointing to the printed option.

### Statistical analyses

An exploratory data analysis was carried out, checking for atypical values and determining the distribution of the continuous quantitative variables using the Shapiro-Wilk test. Descriptive statistics were estimated using the mean and standard deviation (SD) for continuous variables with a normal distribution and the median and interquartile range (IQR) for variables with a biased distribution. In the case of categorical variables, the relative and absolute frequencies were obtained.

Then, survival models were used. These models were used considering that the response variable *discontinuing the use of the hearing aid* that was measured throughout the follow-up period was therefore related to the time (in months) in which participants stopped using the hearing aid. This technique allowed participants to be incorporated into the study at different times, adjusting to the way in which hearing aids were provided (monthly). This was also useful due to the fact that censure existed. This is because when the study ended, some participants were still using their hearing aids.

Discontinuing the use of the hearing aid was expressed using metrics of time passing, such as incidence density, and survival and hazard functions. The numerator for the incidence density was the number of people who stopped using the hearing aid during the follow-up period, and its denominator was the sum of the times at risk of doing so, expressed as people-months. This is a way of quantifying the speed of an event’s occurrence – in this case, discontinuing hearing aid use. The survival function was a way of showing the survival distribution for each of the studied variables. On the vertical axis, the probability of continuing to use the hearing aid to a certain point in time (t) was shown. When estimating the hazard function, Flexible Parametric Royston-Parmar Survival Models [29] were used, given the fact that there was no compliance with the proportionality assumption of the semi-parametric hazard models (Cox’s Proportional Risks) and that estimations are more precise [29]. The change in the hazard of discontinuing hearing aid use (binary outcome) according to the variables of interest (univariate models) adjusted by the covariates (multivariate models) was estimated. Covariates included age, gender, self-perceived state of health, self-perceived hearing problems, satisfaction with the hearing aid and either income or education quintiles. Reparameterization using quartiles or quintiles is a way to detect nonlinear trends in the predictor variables [30]. Quartiles or quintiles were used due to the fact that sample sizes were generated after the overall sample was divided. Grouping in quartiles or quintiles that originated homogeneous sample sizes were chosen.

Due to collinearity, the education and income quintiles were not simultaneously included in the adjusted models. Possible significant differences across categories of ordinal variables (overall effect) were obtained using a Wald test [31].

## Results

### Descriptive statistics

A total of 355 older adults with hearing aids fitted at La Florida Hospital were visited at their homes. Their ages varied between 65 and 85 years (average age: 74.9 years, see Table 1). Gender proportions were similar. The median number of years of education was 9 (interquartile range [IQR] = 6). Most of the participants (35%) reported reaching up to six years of formal education (primary education). Almost 25% reported working at least an hour a day. Their median monthly income was 200,000 (IQR = 150,000) Chilean pesos, ranging between $0 and $2223 USD (see Table 1).

**Table 1:** Socio-demographic variables for the sample of older adults fitted with a hearing aid at La Florida Hospital (*n* = 355)

	Average/median or percentage
Age (average years and SD)	74.9 (5.9)
Gender	
Women	182 (51.3%)
Years of education (median and IQR)	9 (6)
Primary	126 (35.5%)
Secondary	89 (25.1%)
Technical, commercial, industrial, other.	29 (8.1%)
Has a paid job	79 (22.3%)
Income (median and IQR in Chilean pesos)	200,000 (150,000)

*SD* Standard deviation

*IQR* Interquartile range

**Fig. 1:**
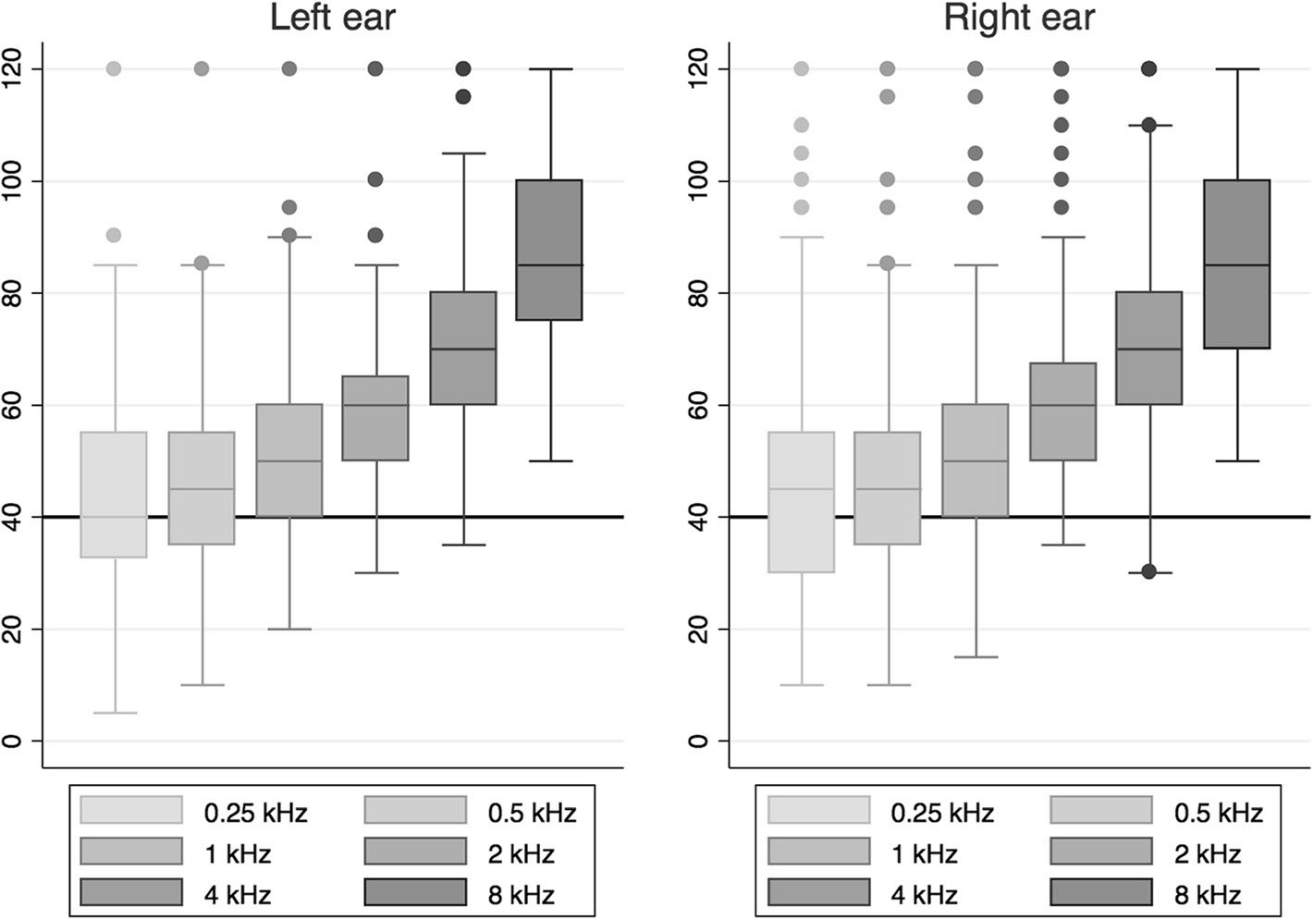
Hearing thresholds in dB HL for both ears in patients fitted with a hearing aid at La Florida Hospital. The horizontal line denotes the criterion for hearing aid prescription (i.e. 40 dB HL) according to the Chilean Ministry of Health’s clinical guidelines

All the participants presented with bilateral sensorineural hearing loss of different degrees. The right ear pure-tone threshold average (500–4000 Hz) was 57.3 dB HL (range: 35.0–112.5 dB HL) and 55.6 dB HL for the left ear (range: 37.5–90.0 dB HL; see Fig. 1).

A total of 85.6% of the participants reported that they could not hear normally without using a hearing aid. In addition, 70.6% reported they could not watch television at a volume that was acceptable to others, and 50.4% could not follow a conversation involving three or more people without wearing a hearing aid (see Table 2). The average score on the S-AIADH was 3.0 points (see Table 2).

**Table 2:** Self-perception of hearing problems, auditory disability, and pure-tone average in older adults fitted with a hearing aid at La Florida Hospital (*n* = 355)

Questions about self-perceived hearing problems	Proportion or average (95% CI)
1. Do you think you hear normally in both ears?	
Negative reply (%)	85.6 (81.6–88.9)
2. Can you watch a TV programme at a volume that is acceptable to others?	
Negative reply (%)	70.6 (65.6–75.2)
3. Can you have a conversation with three or more people?	
Negative reply (%)	50.4 (45.2–55.6)
Auditory disability	
S-AIADH (average score)	3.0 (3.0–3.1)
Pure-tone average (500, 1000, 2000, 400 Hz)	
Right ear (dB HL)	57.3 (54.9–59.8)
Left ear (dB HL)	55.6 (53.3–57.8)

Values are expressed as average or relative frequencies as appropriate, with a 95% CI

### Discontinuation of hearing aid use

Metrics related to how long it took the participants stop using the hearing aid were estimated. These metrics included the incidence density and survival and hazard functions. The accumulated incidence or percentage of discontinuation of the use of the hearing aid at the end of the follow-up was 21.7% (95% CI [17.7–26.3]). The incidence density was 1.31 per 100 people-months (1.31 new cases discontinuing hearing aid use per 100 people per month) in a maximum of 30 months of follow-up after the hearing aid was fitted. Table 3 shows the main reasons for discontinuing the use of the device, with the most commonly mentioned reasons being no/poor benefit and noisy situations are disturbing, each with 18.2% (95% CI [10.9–28.7]). The ‘other reasons’ option was reported at 53.2% (95% CI [41.9–64.3]). These reasons included problems handling the hearing aid, malfunction of the hearing aid, sound quality and loss of the hearing aid.

**Table 3:** Frequency of use, discontinuation of use, reasons for stopping the use of, and satisfaction with the hearing aid

Variables	Proportion (95% CI)
Frequency of weekly use (*n* = 355, 100%)	
Every day	42.3 (37.2–47.5)
Almost every day (at least 5 days a week)	13.5 (10.3–17.5)
Some days (1–4 days a week)	15.8 (12.3–20.0)
Almost never	6.8 (4.6–9.9)
Never	21.7 (17.7–26.3)
Frequency of daily use (*n* = 278, 100%)	
All day	48.6 (42.7–54.5)
A large part of the day	16.2 (12.3–21.0)
Half a day	11.9 (8.5–16.3)
Less than half a day	5.8 (3.5–9.2)
Only for short periods	17.6 (13.6–22.6)
Reasons for discontinuing the use of hearing aid (*n* = 77 non users)^a^	
No/poor benefit	18.2 (10.9–28.7)
Noisy situations are disturbing	18.2 (10.9–28.7)
Poor fit and comfort	15.6 (9.0–25.7)
Difficulties using it (for example, controlling the volume)	15.6 (9.0–25.7)
Poor sound quality	5.2 (1.9–13.3)
Negative side effects (for example, rashes, itching, pain, build-up of wax)	5.2 (1.9–13.3)
No need	2.6 (0.6–10.1)
Other reasons	53.2 (41.9–64.3)
Satisfaction with the hearing aid (*n* = 355, 100%)	
Very satisfied	46.7 (41.5–52.0)
Rather satisfied	27.8 (23.3–32.8)
Rather dissatisfied	12.9 (9.8–16.9)
Very dissatisfied	12.6 (9.5–16.5)

^a^The patient could select more than one option

**Fig. 2:**
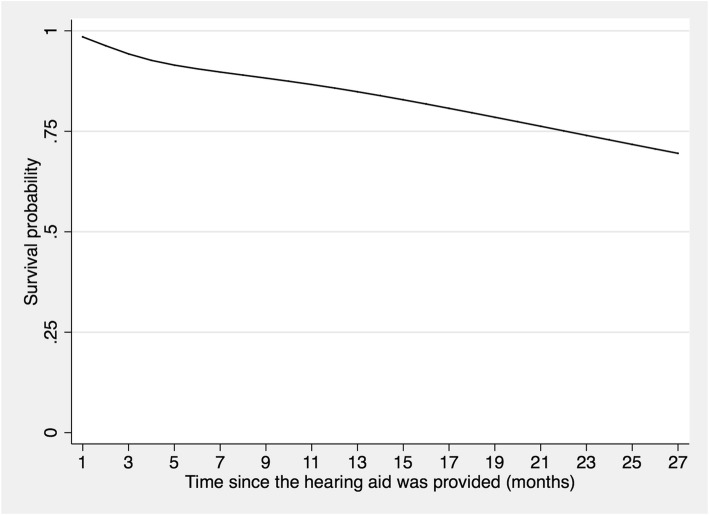
Survival function obtained using flexible parametric models for older adults fitted with a hearing aid at La Florida Hospital

The survival function was 0.96 (95% CI [0.94–0.98]) at the first month of follow-up; 0.85 (95% CI [0.81–0.88]) at 12 months; and 0.73 (95% CI [0.67–0.78]) at 24 months (see Fig. 2). Based on these results, the greatest number of people stopped using their hearing aid in the first 5–6 months. In the following months, a lesser number of participants stopped using their hearing aid.

### Socio-demographic variables associated with discontinuing the use of the hearing aid

In both the univariate and multivariate flexible parametric survival analysis, the demographic variables (i.e. gender and age) were not associated with discontinuing the use of the hearing aid (see Table 4). Age was also categorised in quartiles in case there was a non-linear effect, but no differences were observed among the different quartiles or in the tendency to stop using the hearing aid (*Wald - X*^*2*^ = 4.55; *p =* 0.208). In the univariate model, a significant association between worse self-reported state of health and discontinuing hearing aid use was observed. However, when controlling for covariates, such an association was no longer significant (see Table 4).

**Table 4:** Estimations of the effect (i.e. Hazard Ratio) of socio-demographic variables on discontinuation of hearing aid use

Independent variables	Univariate Hazard Ratio (95% CI)	Adjusted Hazard Ratio^a^ (95% CI)
Gender		
Male	Reference	Reference
Female	1.51 (0.96–2.38)	1.30 (0.78–2.18)
Age	1.04 (1.00–1.07)	1.00 (0.95–1.04)
Education (years)	0.94 (0.89–0.99)*	0.96 (0.90–1.02)
Education (quintiles)		
1° Quintile	Reference	Reference
2° Quintile	0.65 (0.35–1.24)	0.73 (0.36–1.50)
3° Quintile	0.78 (0.41–1.49)	1.45 (0.70–2.97)
4° Quintile	0.41 (0.19–0.88)*	0.48 (0.20–1.16)
5° Quintile	0.50 (0.24–1.01)	0.55 (0.26–1.18)
Income (in Chilean pesos)	1.00 (0.99–1.00)	1.00 (0.99–1.00)
Income (quintiles)		
1° Quintile	Reference	Reference
2° Quintile	0.68 (0.34–1.37)	0.82 (0.40–1.70)
3° Quintile	1.32 (0.73–2.38)	1.35 (0.71–2.59)
4° Quintile	0.40 (0.19–0.86)*	0.65 (0.29–1.47)
5° Quintile	0.49 (0.22–1.12)	0.39 (0.16–0.96)*

^a^ Models for which each independent variable was adjusted by age, gender, self-perceived state of health and auditory problems, satisfaction with the hearing aid and income quintiles. Education and income quintiles were not simultaneously included in the adjusted models due to collinearity

Statistically significant effects are highlighted in bold: *p < 0.05

Differences were observed according to years of education and when this variable was divided into quintiles (Table 4). However, when controlling for covariates, in both cases the effect was no longer significant. In the univariate model for each year of education, the hazard of discontinuation of hearing aid use decreased by 1.06 (HR: 0.94; 95% CI [0.89–0.99]). When categorising using quintiles, it was 2.43 less likely that an older adult in the fourth education quintile has stopped using the hearing aid than someone with less education. In the multivariate model, this quintile was not significantly associated with the hazard of discontinuation of hearing aid use (*p =* 0.106). The same was true when considering the overall effect of the variable (*p = 0.069*).

**Fig. 3:**
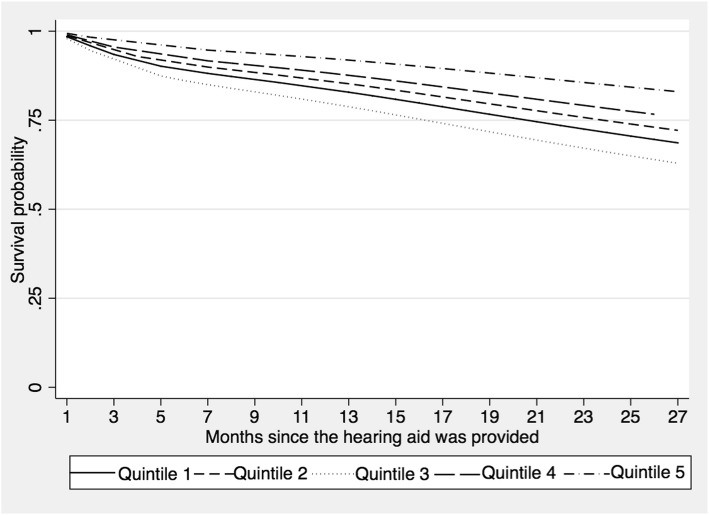
Survival function by economic income quintiles (fifth quintile has the highest income) of older adults fitted with a hearing aid at La Florida Hospital

In the univariate model, the fourth income quintile was significantly different than the other quintiles. Participants in this quintile were 2.50 times less likely to stop using the hearing aid compared to those in the first quintile (lower income). However, in the multivariate model, it was observed that participants in the fifth quintile (higher income) were 2.56 times less likely to discontinue hearing aid use as compared to participants in the first quintile (see Fig. 3).

### Auditory variables associated with the hazard of discontinuing hearing aid use

No significant association between the hazard of discontinuing hearing aid use and self-reported auditory disability (HR: 0.75; 95% CI [0.52–1.07]; see Table 5) was found. Nor was there any effect on separating the S-AIADH’s score into quartiles (*Wald – X*^*2*^ = 2.57; *p =* 0.46). When separating the 25th percentile with the greatest difficulties, fewer older adults tended to stop using their hearing aid (HR: 0.68; 95% CI [0.42–1.10]).

**Table 5:** Estimations of effect (i.e. Hazard Ratio) of self-perceived general state of health along with audiological variables on discontinuation of hearing aid use

	Univariate^a^ Hazard Ratio (95%CI)	Adjusted^a,b^ Hazard Ratio (95%CI)
Self-Perceived general state of health	1.44 (1.08–1.92)*	0.98 (0.72**–**1.33)
Auditory disability (S-AIADH average score)	0.75 (0.52–1.07)	
Do you think you hear normally in both ears?	2.13 (1.24–3.66)**	2.62 (1.44–4.78)***
Can you watch a TV programme at a volume that is acceptable to others?	0.78 (0.49–1.26)	
Can you have a conversation with three or more people?	0.73 (0.46–1.15)	
Pure tone threshold average in the fitted ear	0.96 (0.93–1.00)	
Are you happy with your hearing aid?		
Very satisfied	Reference	Reference
Rather satisfied	3.07 (1.41–6.70)**	2.39 (1.06–5.36)*
Rather dissatisfied	6.17 (2.77–13.74)***	6.55 (2.91–14.74)***
Very dissatisfied	17.18 (8.36–35.30)***	20.86 (9.43–46.15)***

^a^In the case of ordinal variables, overall significance was measured using the Wald Test

^b^Completely adjusted model, which includes age, gender, self-perceived general state of health and hearing problems, satisfaction with the hearing aid and income quintiles

Statistically significant effects are highlighted in bold: *p < 0.05; **p < 0.01; ***p < 0.001

**Fig. 4:**
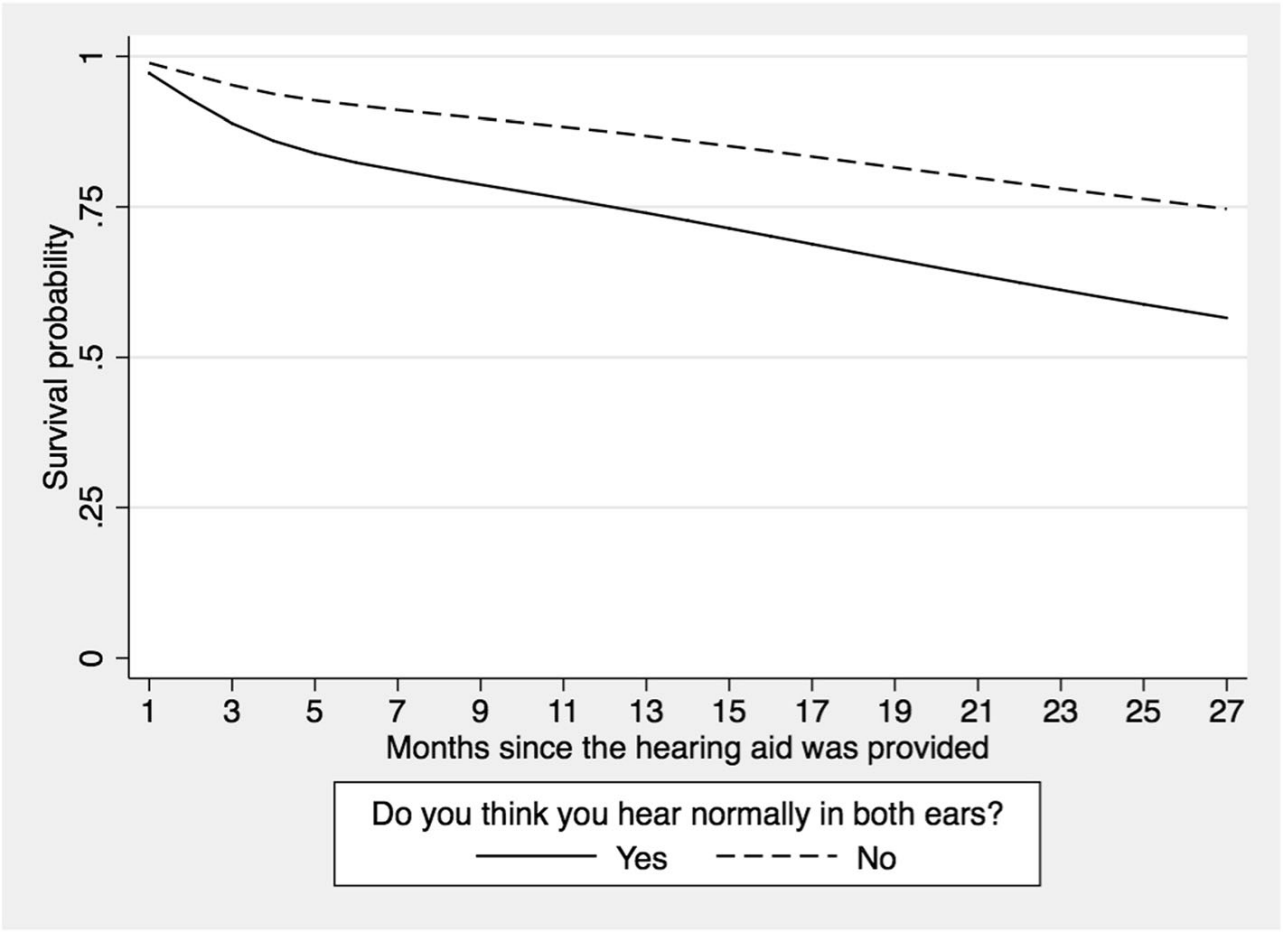
Survival function according to self-perceived auditory problems in older adults fitted with a hearing aid at La Florida Hospital

The perceived hearing problem (determined using the question *Thinking about your hearing without a hearing aid, do you think you hear normally in both ears?)* was significantly associated with discontinued use of the hearing aid (HR: 2.13; 95% CI [1.25–3.66]) in both the univariate and multivariate models (HR: 2.62; 95% CI [1.44–4.78]). Participants who indicated that they could hear normally in both ears were 2.6 times more likely to stop using their hearing aid than those who reported that their hearing was not normal. In Fig. 4, participants who did not report hearing problems were more likely to stop using the hearing aid as time progressed from when the hearing aid was first provided.

It is notable that not recognising a hearing problem (*Do you think you hear normally in both ears?)* is positively associated (rho: 0.18; *p* < 0.05) with auditory disability (S-AIADH). In other words, participants who do not recognise that they have a hearing problem have lesser auditory disability (with a higher score on the S-AIADH for *lesser disability).* In the case of audiometric thresholds, these are negatively related to scores on the S-AIADH (rho: 0.23; *p <* 0.01) and to not recognising the hearing problem (rho: 0.23; *p <* 0.01). The higher the audiometric threshold, the more likely the self-perception of not hearing normally and having a greater auditory disability in daily life are observed.

Participants who reported other hearing problems (i.e. hearing problems in everyday situations in the ENS questionnaire) were not more likely to stop using the hearing aid compared to those who did not self-report hearing problems in daily life (see Table 5). This is also true for the association between discontinuation of hearing aid use and pure-tone threshold in the fitted ear (HR: 0.96; 95% CI [0.93–1.00]; see Table 5).

**Fig. 5:**
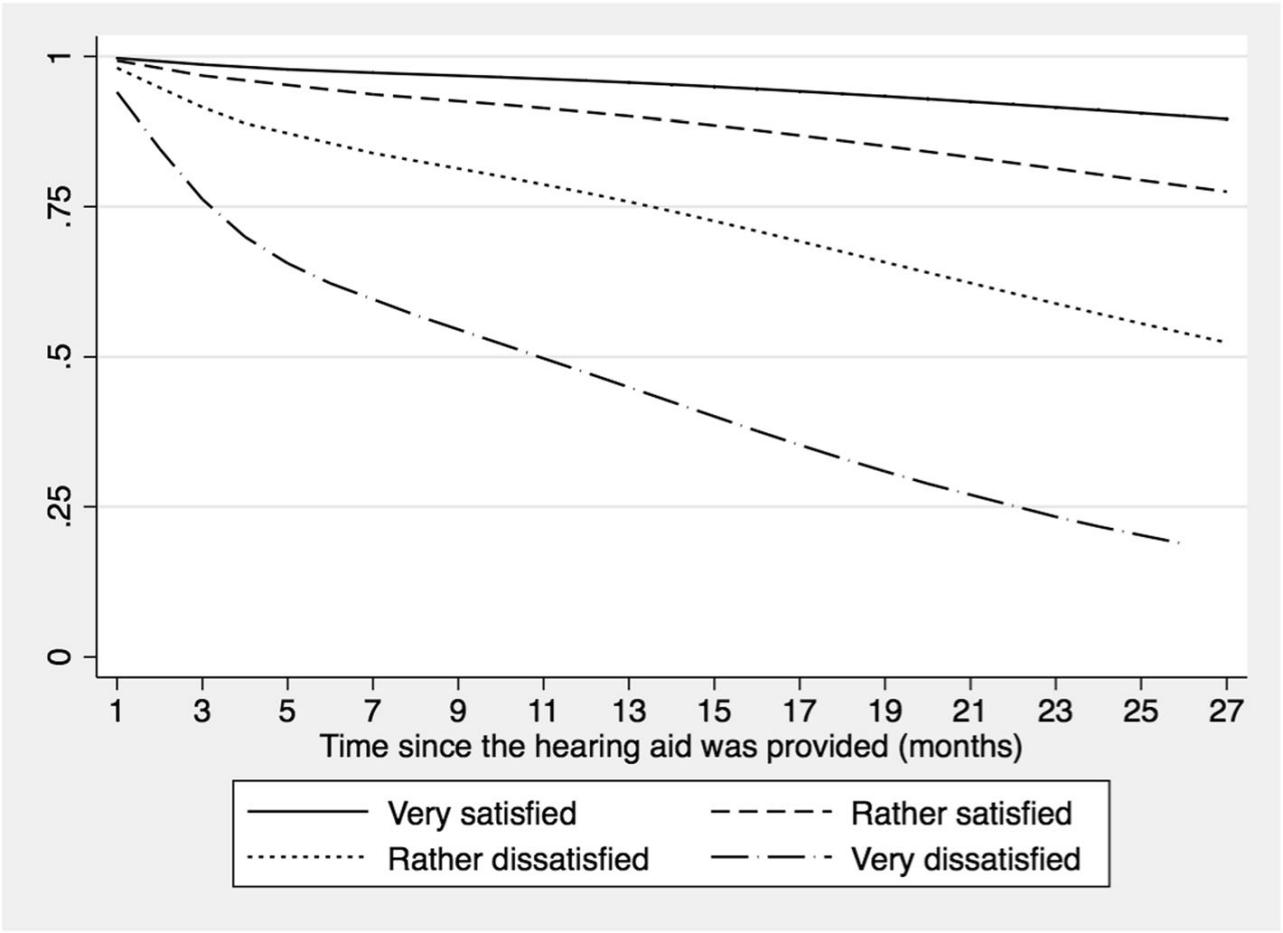
Survival function according to satisfaction with the use of the hearing aid in older adults fitted at La Florida Hospital

Finally, the lower the satisfaction with the hearing aid, the greater the hazard of discontinuing the use of the device (*Wald - X*^*2*^ = 68.69; *p <* 0.001). In Fig. 5, the difference between these categories can be observed. Participants who reported being very dissatisfied with the hearing aid were 20.9 times more likely to stop using their hearing aid (95% CI [9.43–46.15]) than the other groups (see Table 5).

## Discussion

### Discontinuation of hearing aid use

One of the aims of this study was to determine the percentage of older adults who had been fitted with a hearing aid at a public hospital in Chile who discontinued using their hearing aid. Based on the question about weekly use of the hearing aid used by Bertoli et al. [28], the accumulated incidence of discontinuation of hearing aid use was estimated at 21.7% in a maximum of 30 months of follow-up. The most reported reasons for stopping the use of the device were no/poor benefit and disturbing noisy situations. This is in agreement with a population-based study in the USA [32] and a literature review carried out by McCormack and Fortnum [7]. In the case of the study carried out in the USA, people perceived little benefit from the hearing aid because it amplified sounds other than words and no improvements were noted in acoustically complex environments [32].

It is important to mention that the ‘other reasons’ option was selected by more than 50% of participants, who provided specific information about the reasons they stopped using their hearing aid. A large number of participants reported difficulties handling the hearing aid as the main reason. Other reasons included poor sound quality, malfunctioning of the device and loss of the device. These aspects, with the exception of loss of the device, are supposed to be part of the contents addressed during the follow-up sessions provided by the hearing aid dispenser. A total of 30.1% (95% CI [24.8–36.0]) of the older adults attended none or only one of such follow-up sessions. We hypothesise that mobility problems or difficulties travelling to the hospital may be the main reason for participants not attending follow-up sessions. Other possible reasons that may be associated with discontinuing hearing aid use may relate to age-related manual dexterity and sight problems. Also, we hypothesise that participants were not aware that the hearing aid can be replaced free of charge if it does not work properly. This should be addressed in one of the follow-up sessions that participants who experienced a malfunctioning hearing aid probably did not attend.

Gianopoulos et al. [33] in the United Kingdom observed that an important number of the difficulties experienced by patients fitted for the first time with a hearing aid could be solved by adequate follow-up. As a result, it is clear that the Chilean public health system’s third-party follow-up system needs to be evaluated. This should be done with the aim of improving the way in which appointments where hearing aids are adjusted and users are taught how to handle them are performed, together with the need to incorporate auditory rehabilitation after fitting.

The rate of discontinuation of hearing aid use in this study is higher than that reported in Switzerland (3%) using the same instrument but in a different language [28]. Three aspects related to intervention programmes in older adults may explain these differences: (1) the way in which the candidates are chosen to be fitted with hearing aids, (2) the hearing aid technology and (3) how follow-up appointments with the patient are performed. In Switzerland, there is close collaboration between specialists and the companies that provide the hearing aids, and not just as far as audiometric criteria for choosing the candidates is concerned [28]. In addition, the hearing aids use sophisticated technology that improves sound quality, and constant advice and support is included in the follow-up sessions [28]. Although some of these aspects are included in the Chilean system, the fitting process depends on the company that obtained the contract to provide the hearing aids creating heterogeneity among the hospitals. The aforementioned programme guarantees a follow-up appointment a year after fitting [14]. This is certainly too late. Moreover, it is only recommended that the company make follow-up appointments, and is not verified. The hospital only keeps records of the hearing aids fitted, with no further information such as the number of appointments patients attend, the presence of problems with using/looking after the hearing aid and/or other difficulties, or the percentage of patients who stop using the hearing aid.

Regarding the time frame when the discontinuation of hearing aid use occurs, the only prior study is from South Korea [8], where hearing aids are not subsidised by the state. In addition, the study included a wide range of people, including patients with unilateral hearing loss. In South Korea, the percentage of older people who stopped using the hearing aid was greater (31.9%) than what we found. However, the follow-up time period was considerably longer at 4.9 years from when the hearing aid was fitted for the first time, up to a maximum of 13.9 years. There was a noticeable increase in discontinuation of hearing aid use during the first year post-fitting and then 4–5 years afterwards. In the present research, there was a peak in discontinuation of hearing aid use 5–6 months after it was fitted (at 6 months, 9.4% stopped using it), increasing considerably to 14.2% at 12 months and 20.4% at 18 months. The follow-up time of this group should be increased in order to determine whether there is any subsequent peak in discontinuation of use. The differences between the studies might be explained by the timescale used (months in the case of this research and years by Lee and Noh [8]) and by the already mentioned characteristics of the selected context and sample.

### Socio-demographic and auditory variables associated with discontinued hearing aid use

The second aim was to determine the effect of self-perceived hearing problems, the audiometric threshold, auditory disability, hearing aid satisfaction and socio-demographic variables on the hazard of discontinuing hearing aid use in Chilean older adults. Differences were detected according to income quintile, with the fifth quintile (with higher income) being almost three times less likely to stop using their hearing aid compared to the first. This is in agreement with some studies carried out in developed countries. In the United States, Garstecki and Erler [34] observed that not having the income necessary to cover daily needs was related to stopping the use of the hearing aid. In Finland, Lupsakko et al. [3] determined that discontinuation of hearing aid use was associated with a lower income, as well as other variables such as cognitive capacity and difficulty carrying out everyday activities. An association between income level and stopping hearing aid use is expected in a system whose follow-up period is short and is limited to certain benefits, with the patient having to spend their own money to maintain the hearing aid. These expenses involve batteries, changing the connecting tube between the ear mould and the hearing aid, materials necessary to clean the device and eventual circuit/electronic maintenance. Therefore, we hypothesise the reason for the discontinuation of hearing aid use among people from quintiles with lower incomes is mainly due to the cost of hearing aid maintenance.

Despite the fact that 100% of this study sample had hearing loss based on the audiogram, 14.4% reported that they heard normally in both ears without a hearing aid. It was observed that those who were unaware of their hearing problems were almost three times more likely to stop using their hearing aid than those who were. The survival curve of this last group shows that the discontinuation of hearing aid use increases progressively from the time the hearing aid is fitted. Similarly, Garstecki and Erler [34] found an association between male older adults who recognised that they had a hearing problem and use of the hearing aid. In the present study, no association between gender and discontinuation of hearing aid use was found. In addition, studies conducted in developed countries have found an association between recognition of hearing problems and the number of hours hearing aids are used [35–37]. Hours of hearing aid use were not explored in this investigation.

The fact that a percentage who did not perceive they had hearing problems received a hearing aid anyway is explained by the way in which the Chilean public healthcare system works. Although the clinical practice guidelines generated by the Chilean Ministry of Health recommend using the Shortened Hearing Handicap Inventory for the Elderly questionnaire (HHIE-S) as well as pure-tone audiometry, in practice only the pure-tone average criterion is used (bilateral pure-tone average ≥ 40 dB HL). Therefore, an older adult who presents with bilateral moderate hearing loss, no matter what he or she self-reports, is still fitted with a hearing aid. Stephens et al. [38] recommended that both self-reported hearing problems and pure-tone average should be considered to select hearing aid candidates. If there are discrepancies between the two, the patient and his or her family should be counselled before the hearing aid is fitted. Social pressure from family members and/or friends could be a variable that influences consulting for hearing problems. Thus, older adults who are not aware of their hearing problems and who are still fitted with a hearing aid subsequently stop using it.

The average score on the S-AIADH was significantly higher (an average difference of 0.4 points; 95% CI [0.3–0.6]; *p* < 0.001) than the score obtained by the S-AIADH validation study (mean = 2.6; 95% CI [2.4–2.8]). Given that a higher score in this instrument means less disability, this sample involves fewer people with hearing disabilities than those in the validation report. In this study, an association between auditory disability in daily life and satisfaction with the hearing aid was observed. Participants with lower self-perceived auditory disability seemed less satisfied with their hearing aids. This agrees with prior studies conducted in developed countries that found the greater the auditory difficulties, the greater the use of and satisfaction achieved with the hearing aid [39–41]. It is worth determining the variables associated with satisfaction because in this research, this was strongly associated with discontinued hearing aid use.

Finally, although the association between the pure-tone average in the fitted ear and discontinuation of hearing aid use was not significant, it cannot be completely ruled out. The audiometric threshold may have a mediated effect – through the perception of auditory difficulties – on discontinued use of the hearing aid. In this research, the percentage of participants who stopped using their hearing aid is too low to test this hypothesis.

## Limitations and projections

One of the main limitations in this study is a possible memory bias when participants had to recall the month in which they stopped using the hearing aid. To minimise this, the interviewers were trained so that during the interview, they could give clues to help the older adult remember such an event. Such clues included references to significant dates (for example, birthdays, the Chilean national holiday or Christmas). In addition, due to the fact that some prospective candidates refused to take part in the study (n = 35) and that the sample included only participants aged between 65 and 85 fitted with a hearing aid at a specific clinic, extrapolating the results for the total population of older adults who are fitted with hearing aids in the Chilean public healthcare system may not be adequate. However, the sample of older adults investigated in this research comes from a community of older adults who live in a district (i.e. La Florida) of the city of Santiago that has similar characteristics to the general older Chilean elderly population. For example, 90% of older adults in this district are users of the public healthcare system. This percentage is similar to the overall population of older adults in Chile who utilise this system. In addition, information about only gender and age for older adults who did not choose to participate in the study and participants who were excluded due to the exclusion criteria was available. It should be noted that these variables were not associated with discontinuation of hearing aid use in this study. Therefore, this lack of information did not allow us to compare the variables associated with discontinued use between included and excluded participants. A posteriori analysis of the sensitivity of the estimations was not possible for the same reason.

Another limitation is that satisfaction was measured using a unique question. Although there are questionnaires for this (the Satisfaction with Amplification in Daily Life, or SALD [42], for example), these include a large number of items, making clinical application – and particularly, application in epidemiological studies such as this one – difficult. On the other hand, it could be worthwhile to study the influence of other types of prejudice that affect satisfaction. For example, a recent study in Brazil reported that believing the hearing aid provided by the public system is of poor quality affects satisfaction [16].

## Conclusions

The levels of discontinuation of hearing aid use reached 21.7% in a maximum of 30 months of retrospective follow-up. The greatest number of people who stopped using the hearing aid did so in the first 5–6 months, with the effect becoming more gradual after that. Socio-demographic variables such as economic income, self-perceived hearing loss and satisfaction with the hearing aid were significantly associated with discontinued use of the device. Self-perception of hearing loss should be considered when choosing candidates for free programmes providing hearing aids for older adults in South America.

## Data Availability

The dataset used and analysed during the current study is available from the corresponding author on reasonable request.

## Abbreviations

95% CI: 95% confidence interval
dB: Decibel
ENADEAM: Encuesta Nacional de Dependencia de las Personas Mayores or Chilean National Survey on Dependency in Older Adults
ENS: Encuesta Nacional de Salud de Chile or Chilean National Health Survey
HL: Hearing level
HR: Hazard Ratio
ISO: International Organization for Standardization
MMSE: Mini-Mental State Test Examination
S-AIADH: Spanish version of the Amsterdam Inventory for Auditory Disability and Handicap
SALD: Satisfaction with Amplification in Daily Life
SL: Sensation level
USD: United States dollar

## References

[1] Dillon H, Birtles G, Lovegrove R (1999). Measuring the outcomes of a national rehabilitation program. Normative data for the client oriented scale of improvement (COSI) and the hearing aid Users's questionnaire. J Am Acad Audiol.

[2] Hickson L, Worrall L (2003). Beyond hearing aid fitting: Improving communication for older adults. Int J Audiol.

[3] Lupsakko TA, Kautiainen HJ, Sulkava R (2005). The nonuse of hearing aids in people aged 75 years and over in the city of Kuopio in Finland. Eur Arch Otorhinolaryngol.

[4] Smeeth L, Fletcher AE, Ng ES, Stirling S, Nunes M, Breeze E (2002). Reduced hearing, ownership, and use of hearing aids in elderly people in the UK—the MRC trial of the assessment and Management of Older People in the community: a cross-sectional survey. Lancet.

[5] Ng JH, Loke AY (2015). Determinants of hearing-aid adoption and use among the elderly: a systematic review. Int J Audiol.

[6] Knudsen LV, Oberg M, Nielsen C, Naylor G, Kramer S (2010). Factors influencing help seeking, hearing aid uptake, hearing aid use and satisfaction with hearing aids: a review of the literature. Trends Amplif.

[7] McCormack A, Fortnum H (2013). Why do people fitted with hearing aids not wear them?. Int J Audiol.

[8] Lee DH, Noh H (2015). Prediction of the use of conventional hearing aids in Korean adults with unilateral hearing impairment. Int J Audiol.

[9] United Nations, Department of Economic and Social Affairs, Population Division (2017). World Population Ageing 2017 - Highlights (ST/ESA/SER.A/397).

[10] Ministério da Saúde do Brasil. Política nacional de saúde da pessoa portadora de deficiência. Brasília: Ministério da Saúde; 2009. [Internet site]. [Last accessed on 2018 December 15]. Available from: http://bvsms.saude.gov.br/bvs/publicacoes/politica_nacional_saude_pessoa_deficiencia.pdf

[11] Ministerio de Salud y Protección Social de Colombia. Resolución 5521 del 27 de diciembre de 2013 por la cual define, aclara y actualiza integralmente el Plan Obligatorio de Salud POS. [Last accessed on 2018 December 15]. Available from: https://www.minsalud.gov.co/sites/rid/Lists/BibliotecaDigital/RIDE/DE/DIJ/resolucion-5521-de-2013.pdf

[12] Bastías G. Valdivia G. Reforma de salud en chile; el plan AUGE o régimen de garantías explícitas en salud (GES). su origen y evolución. Boletín escuela de medicina UC, Pontificia Universidad Católica de Chile 2007; 32(2): 51–58.

[13] Gobierno de Chile. Superintendencia de Salud. Problema de salud AUGE N° 56. Hipoacusia Bilateral en personas de 65 años y más que requieren uso de audífono. [Last accessed on 2018 December 15]. Available from: http://www.supersalud.gob.cl/difusion/572/w3-article-3710.html

[14] Ministerio de Salud de Chile. Guía Clínica AUGE: Hipoacusia bilateral en personas de 65 años y más que requieren uso de audífono. Santiago de Chile: Ministerio de Salud. 2013. [Last accessed on 2018 December 15]. Available from: https://www.minsal.cl/sites/default/files/files/Hipoacusiabilateralmayores65agnos.pdf

[15] Fuentes-López E, Fuente A, Cardemil F, Valdivia G, Albala C (2017). Prevalence and associated factors of hearing aid use among older adults in Chile. Int J Audiol.

[16] Cruz M, Lima M, Santos J, Duarte Y, Lebrao M (2013). Hearing aids use among elderly: SABE study – health, wellbeing and aging survey. Audiology – Commun Res.

[17] Concato J, Peduzzi P, Holfold TR (1995). Importance of events per independent variable in proportional hazards analysis. I. Background, goals, and general strategy. J Clin Epidemiol.

[18] Peduzzi P, Concato J, Feinstein AR (1995). Importance of events per independent variable in proportional hazards regression analysis. II. Accuracy and precision of regression estimates. J Clin Epidemiol.

[19] Peduzzi P, Concato J, Kemper E (1996). A simulation study of the number of events per variable in logistic regression analysis. J Clin Epidemiol.

[20] Johnson EE (2013). Prescriptive amplification recommendations for hearing losses with a conductive component and their impact on the required maximum power output: an update with accompanying clinical explanation. J Am Acad Audiol.

[21] Quiroga P, Albala C, Klaasen G (2004). Validation of a screening test for age associated cognitive impairment in Chile. Rev Med Chil.

[22] Servicio Nacional del Adulto Mayor (SENAMA). 2010. Estudio Nacional de la Dependencia en las Personas Mayores. Impresores Gráfica Puerto Madero, Chile. [Last accessed on 2018 December 15]. Available from: http://www.senama.gob.cl/storage/docs/Dependencia-Personas-Mayores-2009.pdf

[23] Dias EG, Andrade FB, Duarte YA, Santos JL, Lebrão ML (2015). Advanced activities of daily living and incidence of cognitive decline in the elderly: the SABE study. Cad Saúde Pública.

[24] Avila-Funes JA, Garant MP, Aguilar-Navarro S (2006). Relationship between determining factors for depressive symptoms and for dietary habits in older adults in Mexico. Rev Panam Salud Publica.

[25] Albala C, Lebrão ML, León Díaz EM, Ham-Chande R, Hennis AJ, Palloni A, Peláez M, Pratts O (2005). The health, well-being, and aging ("SABE") survey: methodology applied and profile of the study population. Rev Panam Salud Publica.

[26] Ministerio de Salud de Chile. Encuesta Nacional de Salud (E.N.S). 2010. [Last accessed on 2018 December 15]. Available from: http://web.minsal.cl/portal/url/item/bcb03d7bc28b64dfe040010165012d23.pdf

[27] Fuente A, McPherson B, Kramer SE, Hormazábal X, Hickson L (2012). Adaptation of the Amsterdam inventory for auditory disability and handicap into Spanish. Disabil Rehabil.

[28] Bertoli S, Staehelin K, Zemp E, Schindler C, Bodme D, Probst R (2009). Survey on hearing aid use and satisfaction in Switzerland and their determinants. Int JAudiol.

[29] Royston P, Lambert PC (2011). Flexible parametric survival analysis using Stata: beyond the Cox model.

[30] May Susanne, Bigelow Carol (2005). Modeling Nonlinear Dose-Response Relationships in Epidemiologic Studies: Statistical Approaches and Practical Challenges. Dose-Response.

[31] Vittinghoff E, Glidden DV, Shiboski SC, McCulloch CE. Regression methods in biostatistics: linear, logistic, survival and repeated measures models. Springer; 2012.

[32] Kochkin S, MarkeTrak V (2000). Why my hearing aids are in the drawer: the consumers’ perspective. Hearing J.

[33] Gianopoulos I, Stephens D, Davis A (2002). Follow up of people fitted with hearing aids after adult hearing screening: the need for support after fitting. BMJ..

[34] Garstecki D, Erler S (1998). Hearing loss, control, and demographic factors influencing hearing aid use among older adults. J Speech Lang Hear Res.

[35] Brooks DN (1989). The effect of attitude on benefit obtained from hearing aids. Br J Audiol.

[36] Jerram J, Purdy S (2001). Technology, expectations, and adjustment to hearing loss: predictors of hearing aid outcome. J Am Acad Audiol.

[37] Brooks DN, Hallam RS (1998). Attitudes to hearing difficulty and hearing aids and the outcome of audiological rehabilitation. Br J Audiol.

[38] Stephens S, Callaghan D, Hogan S, Meredith R, Meredith R, Davis A (1990). Hearing disability in people aged 50-65: effectiveness and acceptability of rehabilitative intervention. Br Med J.

[39] Cox R, Alexander G, Gray G (2007). Personality, hearing problems, and amplification characteristics: contributions to self-report hearing aid outcomes. Ear Hear.

[40] Takahashi G, Martinez C, Beamer SS, Bridges J, Noffsinger D, Sugiura K (2007). Subjective measures of hearing aid benefit and satisfaction in the NIDCD/VA follow-up study. J Am Acad Audiol.

[41] Mulrow C, Tuley M, Aguilar C (1992). Correlates of Succesful hearing aid use in older adults. Ear Hear.

[42] Cox R, Alexander G (1999). Measuring satisfaction with amplification in daily life: the SADL scale. Ear Hear.

